# Effectiveness of Repeated Ketamine Infusions in Treatment Non-Responding Obsessive Compulsive Disorder: a Randomised Controlled Trial

**DOI:** 10.1192/j.eurpsy.2025.273

**Published:** 2025-08-26

**Authors:** L. Hauhnar, V. S. Mehta

**Affiliations:** 1 Psychiatry, All India Institute of Medical Sciences (AIIMS), Gorakhpur; 2 Psychiatry, Central Institute of Psychiatry, Ranchi, India

## Abstract

**Introduction:**

Though glutamate modulators have been increasingly used with some success in cases of OCD resistant to SRIs, there is limited data on the use of ketamine in OCD. There is one study on the use of multiple ketamine infusions in SRI-resistant OCD, but no studies have yet compared the effectiveness of multiple infusions of ketamine with multiple infusions of an active comparator agent. Benzodiazepines are commonly prescribed for OCD despite the lack of recommendations. The current study was a hospital-based prospective, single-blind, randomised controlled trial conducted over a period of one and a half years to compare the effectiveness of multiple infusions of ketamine with those of midazolam.

**Objectives:**

To look into the immediate effects of ketamine infusion in terms of reduction in illness severity in OCD treatment non-responders in comparison to midazolam infusion.

To determine the time-point associated with the largest change in symptom severity in patients receiving repeated ketamine infusions in comparison to midazolam infusions.

To determine the overall proportion of response of OCD treatment non-responders to ketamine infusions in comparison to midazolam infusions.

**Methods:**

In a hospital setting, we compared the effectiveness of 6 sessions of ketamine infusions (0.5 mg/kg body weight) with that of 6 sessions of midazolam infusions (0.045 mg/kg body weight), given on alternate days on a Monday-Wednesday-Friday schedule, in 30 patients with treatment non-responding OCD. Assessments were made using rating scales: DY-BOCS, MADRS, HAM-A, CADSS and SAFTEE.

**Results:**

At 1 hour and 4 hours after the 1st ketamine infusion, 26% of patients achieved treatment response, while none in the midazolam group did so at these time points (Figures 1 and 2). Maximum symptom reduction occurred after the 1st infusion. By the 6th infusion, 40% of ketamine patients achieved treatment response, compared to 20% in the midazolam group. At 4 weeks after the last infusion, only 1 patient (6%) in the ketamine group maintained treatment response, with none in the midazolam group. Overall, the result indicates that the ketamine group showed significant improvement compared to the midazolam group (F=1.541, p=0.048) with a medium effect size (η2=0.056) (Fugure 3). There were no statistically significant differences between the two groups in terms of overall reductions in MADRS.

**Image 1:**

**Figure:**
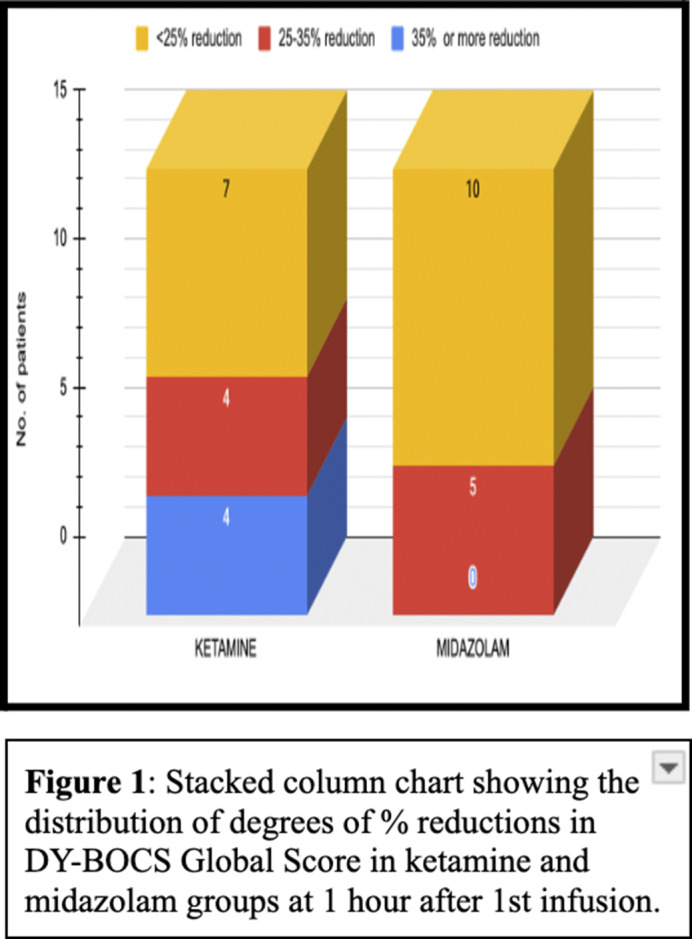

**Image 2:**

**Figure:**
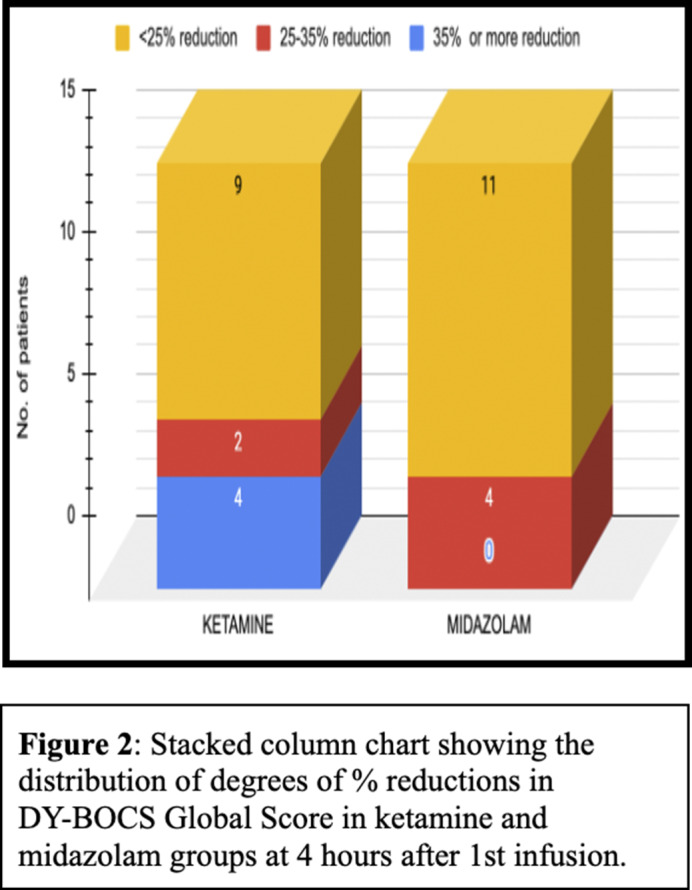

**Image 3:**

**Figure:**
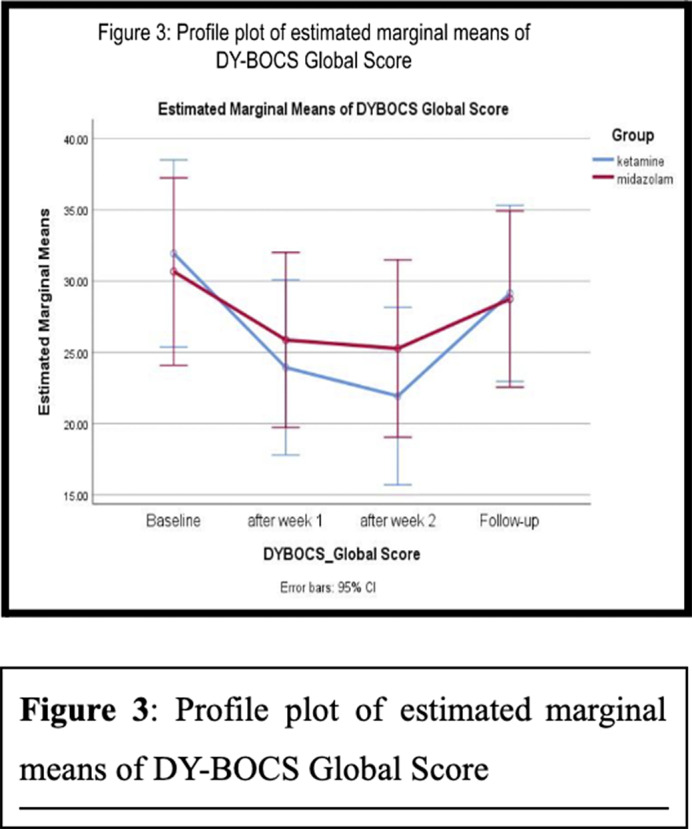

**Conclusions:**

Ketamine, though modest in action, is superior to midazolam. Our findings lie between those of 2 prior studies—that by Bloch et al. (2012), who showed it to be largely ineffective, and that of Rodriguez et al. (2013), who found that half of their participants continued to show response even 1 week after the infusion. The differential overall improvement in DY-BOCS in the ketamine group over the midazolam group was independent of the reductions in depressive symptoms.

**Disclosure of Interest:**

None Declared

